# Quercetin Inhibits AKT Ser473 Phosphorylation and Disrupts AKT–Androgen Receptor Signaling in Castration-Resistant Prostate Cancer Cells

**DOI:** 10.3390/antiox15030393

**Published:** 2026-03-20

**Authors:** Félix Duprat, Sebastián Azócar-Plaza, María Paz Castillo-Cáceres, Yerko Rivas, Javiera Sanzana-Rosas, Paolo Pampaloni, Gabriel Olivas-Henríquez, Jorge Toledo, Jhon López Villa, Romina Bertinat, Nery Jara, Alejandro Vallejos-Almirall, Alexis Salas, Iván González-Chavarría

**Affiliations:** 1 Laboratorio de Lipoproteínas y Cáncer, Departamento de Fisiopatología, Facultad de Ciencias Biológicas, Universidad de Concepción, Concepción 4030000, Chile; 2 Departamento de Química Orgánica, Facultad de Química y de Farmacia, Pontificia Universidad Católica de Chile, Santiago 8320000, Chile; 3 Departamento de Farmacología, Facultad de Ciencias Biológicas, Universidad de Concepción, Concepción 4030000, Chile; 4 Departamento de Ciencia y Tecnología de los Alimentos, Facultad de Farmacia, Universidad de Concepción, Concepción 4030000, Chile

**Keywords:** castration-resistant prostate cancer, quercetin, PI3K/AKT, androgen receptor, PSA, enzalutamide

## Abstract

The progression of prostate cancer to castration-resistant disease (CRPC) remains a clinical challenge in which oxidative stress intersects with the PI3K/AKT–androgen receptor (AR) axis. Quercetin (QRC) is a redox-active dietary flavonol, yet its mechanistic impact on CRPC is incompletely defined. Here, we tested whether QRC suppresses AR output by directly modulating AKT. C4-2B and 22Rv1 CRPC cell lines were treated with increasing QRC concentrations, with or without enzalutamide (Enz). Proliferation and viability were monitored by IncuCyte imaging and SYTOX Green incorporation. AKT phosphorylation (S473), AR phosphorylation (S210/213), AR abundance and localization, and prostate-specific antigen (PSA) secretion were assessed by immunoblotting, immunofluorescence, and dot blot, respectively. Docking and molecular dynamic simulations were performed to identify and evaluate a putative QRC-binding site on AKT. QRC produced a dose-dependent cytostatic effect (IC_50_ 24.37 μM in C4-2B; 21.54 μM in 22Rv1) without marked cell death, reduced pAKT(S473) by up to 80%, decreased pAR(S210/213), and diminished nuclear AR and PSA secretion. Simulations suggested a putative druggable allosteric pocket in the AKT1 N-lobe, with G159 emerging as a potential anchor residue. Enz cotreatment with QRC did not produce additive effects, consistent with a model in which QRC acts upstream of ligand-driven AR activation and thereby limits the incremental benefit of AR antagonism under these conditions. These data support QRC as an AKT–AR axis modulator in CRPC and provide a target engagement framework beyond simple ROS scavenging.

## 1. Introduction

Prostate cancer (PCa) is the second most common malignancy in men worldwide [1]. The androgen receptor (AR)-regulated transcriptional program is essential for maintaining not only normal prostate function but also the growth and survival of malignant prostate tissue, making androgen deprivation therapy (ADT) one of the main therapeutic strategies to restrain PCa progression [2]. ADT reduces testosterone levels, thereby limiting AR stimulation [2]. Unfortunately, 10–20% of these patients eventually relapse and develop resistance against ADT within five years, progressing to castration-resistant prostate cancer (CRPC) [3]. At this stage, the molecular changes in CRPC include AR overexpression, the expression of AR isoforms, AR mutation, intratumoral steroidogenesis that maintains AR activity even under castration conditions and the androgen-independent activation of AR [4,5] by several molecular pathways associated with ERK1/2, SRC/FAK, and AKT [6].

AKT is a serine/threonine kinase and a key effector of PI3K signaling that promotes proliferation, survival, and metabolic remodeling [7,8]. Beyond its essential roles under physiological conditions, AKT hyperactivation is particularly relevant to cancer development and progression [7,8]. Upon PI3K activation, AKT is recruited to the plasma membrane, where it is phosphorylated at threonine 308 (Thr308) within the activation loop by PDK1. Although this event partially activates the kinase, maximal catalytic activity requires an additional phosphorylation at serine 473 (Ser473) mediated by mTORC2 [9,10]. Dual phosphorylation at Thr308 and Ser473 stabilizes AKT in its fully active conformation, thereby enabling the efficient phosphorylation of downstream substrates and amplification of oncogenic signaling [11]. Specifically, AKT has been strongly implicated in prostate carcinogenesis and the development of CRPC [8,12]. Indeed, many mutations in AKT leading to its hyperactivation participate in human PCa [8], with higher levels of both total [13] and S473-phosphorylated AKT being associated with higher Gleason grades in human PCa biopsies [14]. The pathophysiological relevance of this axis is also evidenced by the fact that mice overexpressing AKT1 in the prostate spontaneously developed PCa, mimicking the formation and growth of human PCa [15].

Although anti-androgen therapy inhibits AR activity, the activation of the AR through alternative pathways, such as AKT, can still drive disease progression under castration conditions via ligand-independent AR phosphorylation and activation [8]. The inhibition of AKT has long been of interest in PCa treatment, because a compensatory upregulation of this signaling pathway has been reported following AR inhibition and vice versa [16]. In vitro, the activation of the AKT signaling pathway mediates the phosphorylation at S210/213 and the activation of AR in an androgen-independent manner [17,18]. Furthermore, AR activation induces AKT pathway activation [19]. This positive feedback loop establishes the AKT-AR pathway as a critical target for CRPC.

Natural products, both directly and indirectly as leading compounds for the development of novel drugs, are a critical component of pharmacotherapy [20]. Flavonoids are an important class of natural products, and one of the most common flavonoids is the flavonol quercetin (2-(3′,4′-dihydroxyphenyl)-3,5,7-trihydrochromen-4-one) (QRC) [21]. QRC’s polyphenolic structure confers antioxidant and anti-inflammatory properties, among others, which have proven effective in the treatment of cancer [21,22]. As a redox-active polyphenol, quercetin can act as a radical scavenger and metal chelator, but increasing evidence indicates that flavonoids may also exert non-classical effects by directly engaging signaling proteins, including kinases [23]. Distinguishing redox-dependent effects from direct target engagement is therefore important when considering quercetin (QRC) as a therapeutic scaffold in oncology. Although QRC has been reported to exert anti-carcinogenic effects consistent with the suppression of the PI3K/AKT/mTOR pathway across multiple cancer models, the available evidence suggests that these effects are largely mediated through upstream modulation and do not conclusively demonstrate direct AKT targeting or inhibition [24]. Accordingly, the mechanistic basis remains unresolved, as direct evidence of AKT target engagement (or inhibition of AKT downstream effectors) is still lacking. Moreover, QRC’s role in CRPC and its impact on the AKT–AR signaling axis have not been previously defined.

Here, we tested the hypothesis that quercetin (QRC) suppresses AKT activation (Ser473) and thereby limits AKT-dependent, ligand-independent AR signaling in CRPC. Using the human CRPC cell lines C4-2B and 22Rv1, we leveraged an automated live-cell imaging platform (IncuCyte) to quantify proliferation, membrane integrity cell death, and treatment-induced morphological changes over time. In parallel, we assessed the key readouts of the AKT–AR axis, including AKT Ser473 phosphorylation, AR phosphorylation, nuclear accumulation, and downstream outputs measured as AR mRNA levels (qPCR) and secreted PSA. We further integrated in silico structural modeling to explore a potential allosteric interaction site for QRC within the N-lobe of the AKT1 kinase domain. Finally, we used enzalutamide as a pharmacologic probe to test whether QRC acts upstream of ligand-driven AR signaling, reasoning that a lack of additive effects with enzalutamide would indicate that QRC and enzalutamide act on the same functional AR pathway, consistent with QRC operating upstream of ligand-driven AR activity.

## 2. Materials and Methods

### 2.1. Cell Culture

Human prostate cancer cells C4-2B (#CRL-3315, ATCC, Manassas, VA, USA) and 22Rv1 (#05092802, ECACC, Salisbury, UK) were routinely grown in RPMI 1640 medium, supplemented with 2 mM Glutagro (25-015-CI, Corning, Corning, NY, USA), 10% fetal bovine serum (HC.SV30160.03, Cytiva, Marlborough, MA, USA), and 1% penicillin–streptomycin (HC.SV30010, Cytiva), and maintained at 37 °C with 5% CO_2_ in a humidified incubator. Both cell lines are androgen-independent but can respond to testosterone or DHT by AR activation and are able to secrete PSA [25].

### 2.2. IncuCyte Live-Cell Proliferation and Death Analysis

A total of 5000 cells were seeded in 96-well plates in complete medium. Stocks of QRC (#10005169, Cayman Chemical, Ann Arbor, MI, USA) and enzalutamide (#11596, Cayman Chemical) were dissolved in DMSO. The final DMSO concentration in all assays was 0.1% (*w*/*v*) in the control, QRC, and enzalutamide conditions. A dose–response curve assay was performed for QRC (0, 6.25, 12.5, 25 and 50 μM) alone or in combination with 20 μM enzalutamide for C4-2B or 40 μM enzalutamide for 22Rv1. SYTOX Green (S7020, Invitrogen, Carlsbad, CA, USA) was added to all wells to a final concentration of 30 nM. Subsequently, the plate was analyzed by high-content live-cell imaging using IncuCyte S3 coupled to the cell-by-cell recognition module (CMA Bio Bio, Universidad de Concepción). The IncuCyte was set to take 2 images per well every 1 h with 10× magnification for 0–48 h, in phase contrast and in the FITC channel 498/517 nm. Kinetic death analyses were performed automatically with the cell-by-cell module by continuously dividing the number of dead cells (SYTOX+) by the total number of cells. All viability and cell death analyses were performed in quadruplicate. Time 0 was used as the starting point for each condition, and the control treatments (0 concentration, no drug) were performed with 0.1% DMSO (*w*/*v*). Subsequently, the percentages of cell viability and cell death relative to the control were exported and plotted using GraphPad Prism 8 software.

### 2.3. Antibodies

Mouse anti-phospho-S473-AKT (#4051), rabbit anti-pan AKT (#9272) and rabbit anti-AR (#5153) antibodies were obtained from Cell Signaling Technology Inc. (Danvers, MA, USA). Mouse anti-phosphorylated S210/213 AR (sc-52894), mouse anti-GAPDH (sc-47724) and goat anti-PSA (sc-7638) antibodies were obtained from Santa Cruz Biotechnology Inc. (Dallas, TX, USA). Secondary antibodies for Western blot (Alexa Fluor 790-conjugated donkey anti-mouse IgG (715-625-150) and Alexa Fluor 680-conjugated donkey anti-rabbit IgG (711-655-152)), for dot blot (Alexa Fluor 790-conjugated donkey anti-goat IgG (705-655-147)), and for immunofluorescence (Cy3-conjugated donkey anti-mouse IgG (711-167-003)) were obtained from Jackson ImmunoResearch Laboratories Inc. (West Grove, PA, USA).

### 2.4. Western Blot

A total of 500,000 cells were seeded overnight in RPMI-1640 medium supplemented with 10% FBS. Cells were then serum-starved and maintained in RPMI-1640 medium without serum. After the starvation period, cells were pretreated with quercetin (QRC, 25 μM) for 30 min. Subsequently, a serum pulse of 10% FBS was added to stimulate the activation of the PI3K/AKT-AR signaling pathway. Protein lysates were collected at 1 h and 24 h after serum stimulation for the analysis of AKT, p-AKT, AR, and p-AR by Western blot. Protein extracts were prepared in RIPA buffer supplemented with protease inhibitors (sc-24948, Santa Cruz Biotechnology Inc). Total proteins were quantified with the Pierce™ BCA Protein Assay Kit (#23227 Thermo Fisher Scientific, Waltham, MA, USA) and denatured in loading buffer at 95 °C for 10 min. Subsequently, 30 μg of total proteins was separated in 10% SDS-PAGE and transferred to nitrocellulose membranes. All primary antibodies were diluted in 5% non-fat milk in TBS, except for anti-P-AKT and anti-P-AR which were diluted in 1% BSA/TBS buffer, and membranes were incubated overnight at 4 °C. After three washes, membranes were incubated with secondary antibodies diluted in 5% non-fat milk/TBS buffer at 4 °C for 1 h. The signal was detected using LI-COR Odyssey CLX equipment (LI-COR, Lincoln, NE, USA). All experimental conditions were performed using at least three independent biological replicates.

### 2.5. Dot Blot

PSA was measured in the culture medium (supernatant) collected from treated cells from Western blot experiments (24 h). Total protein concentration in the medium was first quantified using the Pierce™ BCA Protein Assay Kit (#23227, Thermo Fisher Scientific) to allow for the normalization of PSA levels.

For dot blot analysis, 100 μL of culture medium from treated and untreated cells was spotted onto a nitrocellulose membrane. Membranes were then blocked with 5% non-fat milk in TBS for 15 min at room temperature and incubated overnight at 4 °C with a primary antibody against PSA diluted in 5% non-fat milk. After three washes with TBS, membranes were incubated for 1 h at 4 °C with the appropriate secondary antibody diluted in 5% non-fat milk/TBS. The signal was detected using the LI-COR Odyssey CLx imaging system, and PSA fluorescence intensity was normalized to the total protein content present in the corresponding 100 μL of culture medium. All experiments were performed using three independent biological replicates.

### 2.6. Real-Time RT-PCR

A total of 500,000 cells were seeded overnight in RPMI-1640 with 10% FBS, serum-starved, pretreated with quercetin (QRC, 25 μM) for 30 min, and stimulated with a 10% FBS serum pulse to activate PI3K/AKT-AR. Total RNA was extracted using TRIzol reagent, following the manufacturer’s instructions (Life Technologies, Carlsbad, CA, USA). Reverse transcription and real-time PCR for AR (sense 5′-GAACCAGAAACCCTGCAAATGCTC-3′, antisense 5′-GGTGTCCATCTGGCTTTAGGCTTT-3′) were performed with 100 ng of RNA, using the Brilliant II SYBR Green real-time RT-PCR Master Mix 1-Step PCR kit and the AriaMX equipment (both from Agilent Technologies, Santa Clara, CA, USA). The results were analyzed using the 2^−ΔΔCt^ method for relative quantification. The comparative threshold cycle values were normalized for GAPDH mRNA (sense 5′-ACCCCTTCATTGACCTCAAC-3′, antisense 5′-ATGACAAGCTTCCCGTTCTC-3′). All real-time RT-PCR analyses were performed using three independent biological replicates.

### 2.7. Immunocytochemistry

Cells regularly grown in complete RPMI medium with phenol red were washed with PBS twice and then incubated in complete medium without phenol red for at least 48 h. A total of 100,000 cells were seeded overnight in RPMI-1640 with 10% FBS, serum-starved, pretreated with quercetin (QRC, 25 μM) for 30 min, and stimulated with a 10% FBS serum pulse to activate PI3K/AKT-AR. Then, the cells were fixed with cold 4% paraformaldehyde in PBS for 15 min, washed twice and simultaneously blocked and permeabilized with 0.05% Triton X-100 in 1% BSA for 30 min. The primary antibody diluted in 1% BSA was incubated overnight at 4 °C and washed. The secondary antibody diluted in 1% BSA was incubated for 1 h, and the cells were then washed. Nuclear staining was performed using DAPI. The samples were analyzed using a Zeiss LSM780 confocal microscope (Zeiss, Jena, Germany) (CMA Bio Bio, Universidad de Concepción).

### 2.8. Image-Based Densitometry and Fluorescence Quantification

Dot blot and immunofluorescence signal quantification was performed in ImageJ (Version 1.54m) (NIH). Western blot fluorescence signal quantification was performed in Image Studio Version 5.2.5 (LI-COR).

### 2.9. Statistical Analysis

All assays were conducted in at least three independent experiments. Data are presented as the mean ± SEM. Two-group comparisons were performed using Student’s *t*-test (paired when measurements were matched within the same experiment; otherwise, unpaired), and comparisons among multiple groups were analyzed by an ANOVA, as appropriate, followed by post hoc multiple comparison testing. A two-sided *p*-value < 0.05 was considered statistically significant. All statistical analyses and graphs for biological experiments were generated using GraphPad Prism (v8.01).

### 2.10. Molecular Docking and Molecular Dynamics of AKT-QRC Interaction

To explore the interaction between QRC and AKT1, a full-length homology model of human AKT1 (residues 1–480), designated AKT-17, was generated using MODELLER (Version 10.7) [26]. Template selection prioritized high-resolution X-ray crystallographic structures of AKT1 domains to maximize structural accuracy across both the kinase and regulatory regions. The structural templates PDB 6BUU (residues 144–480) [11] and PDB 6HHJ (residues 2–446) [27], selected from the UniProt database [28] (UniProt ID P31749), were analyzed by PDBsum and ProSA-web (Supplementary Figure S1) [29,30] and employed for model construction. Druggability assessment was performed using Fpocket [31]. Molecular docking was conducted using SMINA [32], with the co-crystallized ligand ZXW from the 6BUU structure employed as a reference to guide the placement of QRC within the pocket predicted by Fpocket. A total of 90 independent docking simulations were performed. The optimal pose (QRC-14) was selected based on the hierarchical clustering of RMSD values and proximity to the cluster centroid, as well as population density (Supplementary Figure S2). All scripts and workflows are openly available in a dedicated GitHub repository (https://github.com/Sazocar-P/Molecular_docking_AKT_QUE.git (accessed on 28 May 2025)). To ensure reproducibility and long-term accessibility, this repository was archived in Zenodo under the DOI https://doi.org/10.5281/zenodo.15537643. Subsequent molecular dynamic (MD) simulations were carried out using the AMBER suite, and ligand parameterization was performed with Antechamber using the GAFF2 force field [33]. The AKT-17/QRC-14 complex was solvated in a 50 Å OPC water box with 0.15 M NaCl using TLeap [33]. After energy minimization, heating, and equilibration, a 100-nanosecond production run was performed under NPT conditions using pmemd.cuda [34]. Trajectory analysis was performed using CPPTRAJ (Version V6.18.1) and PyTraj (Version 2.0.6) [35]. System stability and conformational dynamics were evaluated by root mean square deviation (RMSD), root mean square fluctuation (RMSF), and Ramachandran plot analysis. Principal component analysis (PCA) was conducted to identify dominant collective motions. Protein–ligand interactions were further characterized through hydrogen bond analysis (≤3.5 Å), along with the identification of persistent contact residues over the simulation trajectory. To evaluate the contribution of individual residues within the identified binding pocket, in silico alanine scanning was performed using Gnina (Version 1.3.2) [36], a molecular docking tool based on a convolutional neural network scoring function trained to predict binding poses and affinities. Starting from the AKT-17/QRC-14 complex, a set of single-point mutations (G157A, G159A, F160A, G162A, V164A, K179A, and F293A) was introduced. Predicted binding affinities were obtained directly from Gnina’s CNN-based scoring output. Changes in binding energy upon mutation (ΔAffinity) were used to assess the functional contribution of each residue to ligand stabilization.

## 3. Results

### 3.1. QRC Inhibits Proliferation of CRPC Cell Lines

QRC has demonstrated a cytotoxic effect on androgen-sensitive PCa cell lines, such as LNCaP [25]. However, information on the effect of QRC on androgen-independent PCa or CRPC cell lines is scarce.

**Figure 1:**
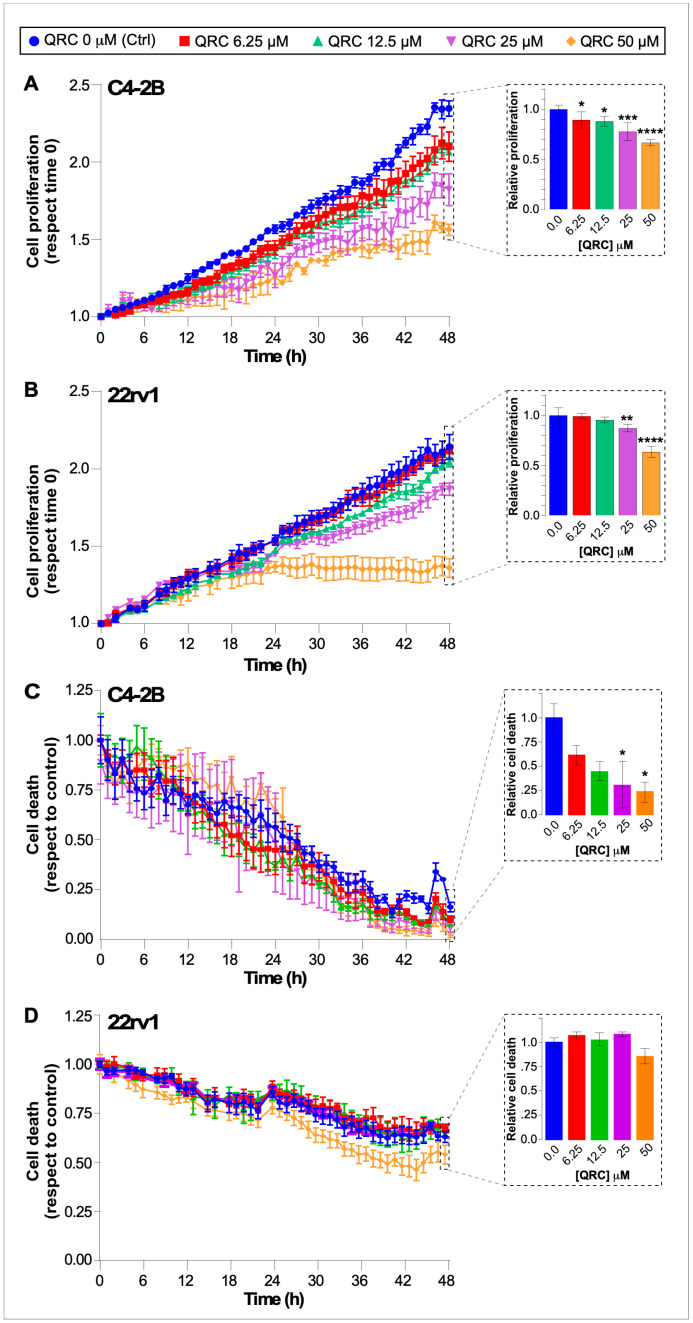
The effect of QRC on the proliferation and cell viability of CRPC cell lines. C4-2B and 22Rv1 cells were cultured in the presence of increasing concentrations of QRC (0, 6.25, 12.5, 25 and 50 μM), and images were taken each hour up to 48 h using the IncuCyte S3 system. The effect of QRC on cell proliferation is shown for C4-2B (**A**) and 22Rv1 (**B**) cells during the 48 h analysis, with the effect at 48 h of treatment presented as a bar plot. The effect of QRC on cell death (based on the incorporation of a live-cell-impermeable nucleic acid stain) is shown for C4-2B (**C**) and 22Rv1 (**D**) cells during the 48 h analysis, with the effect at 48 h of treatment presented as a bar plot. The data represent the means ± SEM of four independent experiments analyzed by a one-way analysis of variance and Dunn’s multiple comparison post-test (**** *p* ≤ 0.0001, *** *p* ≤ 0.001, ** *p* ≤ 0.01, * *p* ≤ 0.05).

First, we determined the IC_50_ of QRC using the crystal violet assay and obtained an IC_50_ of 24.37 μM for C4-2B and 21.54 μM for 22Rv1 (Supplementary Figure S7). Then we performed a dose- and time-dependent study of the effect of QRC on the viability and proliferation of C4-2B and 22Rv1 cell lines using IncuCyte live-cell analysis. To this end, cells were incubated with 0 (Ctrl), 6.25, 12.5, 25 and 50 μM QRC in the presence of SYTOX Green and imaged at time 0 and each hour thereafter for up to 48 h of culture. Proliferation curves show the expected growth of both cell lines in control conditions, with QRC exerting anti-proliferative effects at different time points and concentrations in each cell line (Figure 1A,B). C4-2B cells showed a tendency towards reduced proliferation at all QRC concentrations tested, starting at ~24 h; however, a statistically significant difference was observed at 48 h, with 25 and 50 μM QRC having the greatest effect, reducing proliferation by ~25% (Figure 1A). For 22Rv1 cells, a potent anti-proliferative effect of 50 μM QRC was statistically significant as early as 24 h and remained evident through 48 h of treatment with a reduction of ~40% in proliferation. In contrast, 25 μM QRC produced a rather modest but consistent effect, reducing proliferation by ~15% (Figure 1B).

In parallel, we assessed the impact of QRC on cell viability using SYTOX Green, a membrane-impermeant nucleic acid dye that enters cells only upon the loss of plasma membrane integrity and therefore selectively labels dead cells. Live, healthy cells exclude SYTOX Green. Cell death was quantified over 48 h as the SYTOX Green signal relative to basal (vehicle) conditions. Under these conditions, QRC treatment for 48 h induced only a minor increase in cell death in C4-2B cells (Figure 1C) and had no effect on viability in 22Rv1 cells (Figure 1D) at any tested concentration of QRC. Therefore, we selected 25 μM QRC as the working concentration for the following experiments.

### 3.2. QRC Suppresses AKT Activation and Reduces AR Phosphorylation and Expression in CRPC Cell Lines

The androgen-independent activation of AR is a key mechanism that sustains CRPC progression. Because AKT can phosphorylate AR and enhance its stability and transcriptional output under androgen-deprived conditions [19], we asked whether QRC modulates this signaling node in CRPC models. Although QRC has been reported to dampen PI3K/AKT pathway activity in other malignancies [24], direct evidence for AKT target engagement has been limited. Here, we evaluated the impact of QRC on AKT activation and downstream AR phosphorylation and expression in the CRPC cell lines C4-2B and 22Rv1.

**Figure 2:**
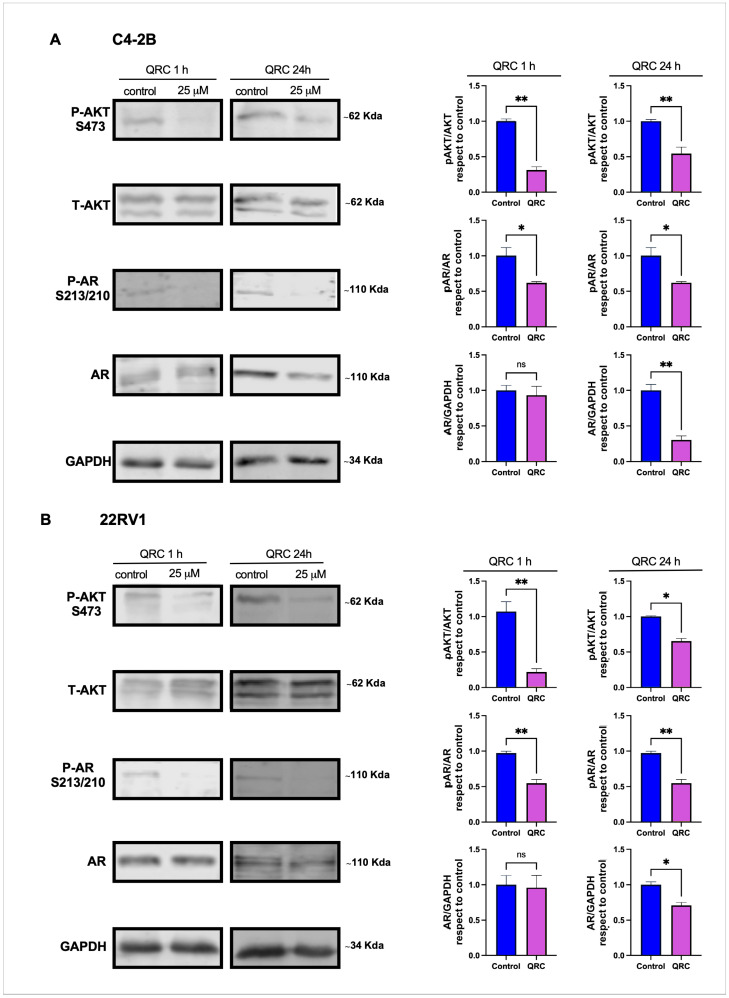
The effect of QRC on AKT activation and AR expression in CRPC cell lines. A Western blot analysis of the levels of AKT phosphorylated at S473 (P-AKT S473), total AKT (T-AKT), AR phosphorylated at S210/213 and total AR (AR) in total protein extracts from C4-2B (**A**) and 22Rv1 (**B**) cells incubated with or without 25 μM QRC for 1 h and 24 h. GAPDH was analyzed as the loading control. The Western blot images were quantified and data presented in plots as follows: pAKT/AKT, pAR/AR, and AR/GAPDH. The data represent the means ± SEM of at least three independent experiments analyzed by Student’s *t*-test (** *p* ≤ 0.01, * *p* ≤ 0.05). ns: not statistically significant.

The basal phosphorylation of AKT at S473 was detectable in both C4-2B and 22Rv1 cells maintained in complete medium containing 10% serum. Treatment with 25 μM QRC significantly reduced pAKT(S473) by ~70% at 1 h and ~50% at 24 h in C4-2B cells (Figure 2A) and by ~80% at 1 h and ~40% at 24 h in 22Rv1 cells (Figure 2B). Total AKT levels were not affected by QRC in either cell line (Figure 2A,B).

AKT has been proposed as a kinase that phosphorylates AR at S210/213, a modification linked to AR stability, nuclear translocation, and transcriptional activity in CRPC [17,18]. We therefore examined AR phosphorylation at S210/213 and total AR levels in C4-2B and 22Rv1 cells treated with 25 μM QRC. AR phosphorylation at S210/213 was detectable under basal conditions in both lines and was reduced by ~50% at 1 h and 24 h following QRC treatment (Figure 2A,B). Total AR levels were not significantly altered at 1 h but decreased by ~50% after 24 h in both cell lines (Figure 2A,B).

### 3.3. Bioinformatic Studies: Molecular Docking and Molecular Dynamic Analysis of AKT-QRC Interaction

**Figure 3:**
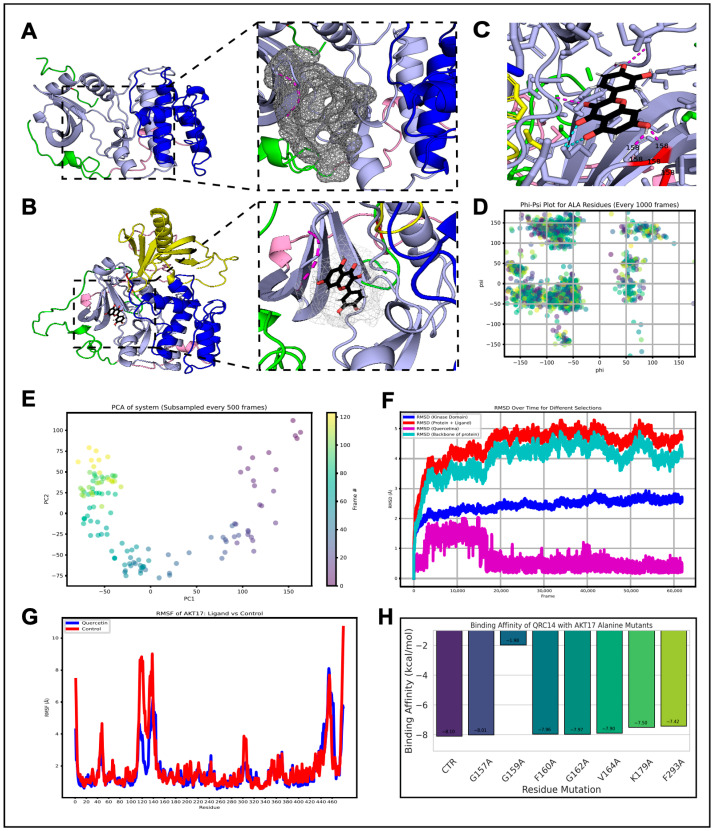
Molecular docking and molecular dynamic analysis of AKT-17/QRC-14 interaction. (**A**) AKT1 6BUU model (residues 144–480) showing kinase N-lobe (149–308, gray), kinase C-lobe (309–409, blue), linker region (410–426, pink), and C-terminal domain (427–480, green); inset: zoom of druggable pocket within kinase domain. (**B**) AKT-17 model showing PH domain (1–113, yellow), linker sequences (114–148 and 410–426, pink), kinase N-lobe (149–308, gray), kinase C-lobe (309–409, blue), and C-terminal domain (427–80, green); inset: QRC-14 (black) positioned in kinase domain pocket. (**C**) Representative docking frame highlighting residues involved in hydrogen bond interactions (hbsc, cyan; hbbb, pink). (**D**) Ramachandran plot of alanine (ALA) residues sampled every 1000 frames during MD simulation of AKT-17/QRC-14 complex. (**E**) Principal component analysis (PCA) of AKT-17/QRC-14 MD trajectory (subsampled every 500 frames). (**F**) RMSD over time for AKT-17/QRC-14 system: kinase domain (blue), protein backbone (cyan), full complex (red), and QRC-14 ligand (purple). (**G**) RMSF per residue for AKT-17/QRC-14 complex (blue) and ligand-free AKT-17 control system (red). (**H**) In silico alanine scanning mutagenesis of binding pocket residues to estimate their contribution to QRC-14 binding stability.

Given that the phosphorylation of S473 is required for the maximal activation of AKT [9], we hypothesized that the inhibitory effect of QRC on AKT phosphorylation at S473 is the result of a direct action. We performed a molecular docking study and an analysis of the molecular dynamics of the putative interaction between QRC and AKT. To this end, we selected the PDB 6BUU structure of AKT1, as it was resolved by X-ray diffraction at 2.4 Å and includes the phosphorylation site S473 [10]. The characterization of the AKT1 model (PDB 6BUU) for molecular docking is presented in more detail in Supplementary Figure S1. The pocket with the highest druggability score (DS = 0.588), which corresponds to a high-confidence binding pocket within the kinase domain in the N-LOBE of AKT1, was selected (Figure 3A–C). The model with the lowest Discrete Optimized Protein Energy, designated as AKT-17, was used for molecular docking studies. In parallel, the QRC ligand to be used was selected by the proximity to the centroid and the largest population (Supplementary Figure S2). QRC conformation number 14 (QRC-14) was selected for the analysis of molecular dynamics with AKT-17. These analyses suggest that QRC may bind to the N-lobe region of the AKT1 kinase domain, potentially exerting an allosteric inhibitory effect on its activation. Molecular dynamics is a computational technique used to simulate the motion and interaction of atoms and molecules in chemical or biological systems. The preparatory steps for our analysis are shown in Supplementary Figures S3–S6. Molecular dynamic (MD) simulations were conducted using the protein topology file (PRMTOP) and the molecular dynamic trajectory file (NC) within the PyTraj environment, employing CPPTRAJ tools [35]. Solvent molecules, including water and Na^+^ and Cl^−^ ions, were removed from both files (PRMTOP and NC) prior to analysis.

Plots for the computational analyses (docking/MD) were generated in Python (Version 3.13.12) within the JupyterLab environment [37]. Molecular structures were visualized using PyMOL (Version 3.1.5.1) [38]. To assess the structural integrity and conformational stability of the simulated system, a Ramachandran plot was constructed, focusing on alanine (ALA) residues due to their critical role in stabilizing secondary structural elements such as α-helices and β-sheets. This analysis showed that most φ/ψ angles remained within the favored regions throughout the trajectory, confirming the good stereochemical quality and structural integrity of the model (Figure 3D). Additionally, principal component analysis (PCA) was performed to elucidate the dominant collective motions within the AKT-17/QRC-14 complex. The PCA projection was color-coded from blue (initial frames) to yellow (final frames), allowing for the visualization of conformational transitions throughout the simulation, revealing a gradual shift in conformational space over time, indicating the presence of collective motions and structural flexibility within the complex (Figure 3E). To evaluate the protein–ligand interaction dynamics, the root mean square deviation (RMSD) was calculated for different structural components, including the AKT-17 backbone, the kinase domain (residues 149–409), the ligand QRC-14, and the entire AKT-17/QRC-14 complex. RMSD analysis demonstrated that both the AKT-17 backbone and its kinase domain reached equilibrium, while QRC-14 maintained a stable binding pose within the druggable pocket, suggesting sustained ligand association (Figure 3F). Additionally, the root mean square fluctuation (RMSF) of the AKT-17/QRC-14 complex and the control system (AKT-17 without QRC) was computed to quantify residue-specific stability throughout the simulation. RMSF analysis revealed a notable reduction in residue-level fluctuations in the ligand-bound system compared to the QRC-free form, particularly in regions surrounding the binding site. Remarkably, the presence of QRC-14 led to a marked stabilization of both the kinase domain hinge region and the PH domain (residues 113–149), which are known to play key roles in the conformational flexibility and activation of AKT1. This observation suggests that QRC may exert a stabilizing effect on the regulatory elements of AKT1, potentially modulating its functional dynamics (Figure 3G). Finally, the formation of hydrogen bonds between AKT-17 and QRC-14 was assessed over the 100 ns MD trajectory. Hydrogen bonds were detected in every frame of the simulation, and statistical analyses were performed to determine their frequency and persistence. Notably, several residues—G157, G159, F160, G162, V164, and F293—remained within 3.5 Å of QRC-14 throughout most of the simulation, suggesting the presence of highly stable and persistent interactions. These residues are located within the kinase domain, further supporting the structural relevance of the identified binding pocket and the robustness of the AKT-17/QRC-14 complex. In silico alanine scanning mutagenesis revealed the differential contributions of individual residues to QRC-14 binding affinity (Figure 3H). The docking affinity for the wild-type AKT-17/QRC-14 complex was −8.10 kcal/mol. Single-point alanine substitutions at residues G157, F160, G162, V164, K179, and F293 resulted in minor decreases in predicted binding affinity, ranging from −7.42 to −8.00 kcal/mol. In contrast, the G159A mutation led to a marked reduction in affinity, yielding a value of −1.98 kcal/mol (Figure 3H). These results suggest distinct roles for residues within the binding interface, with some contributing modestly to ligand stabilization and others having a more pronounced effect on binding strength.

Overall, our detailed analysis proposed that QRC binds directly to the N-LOBE region in the kinase domain of AKT1, suggesting an allosteric inhibitory effect over its activation. Furthermore, the molecular dynamic analysis suggests that this interaction is stable and of biological significance, making QRC a promising lead compound for the inhibition of AKT activity.

### 3.4. QRC Reduces AR Expression, Nuclear Accumulation and PSA Expression in CRPC Cell Lines

**Figure 4:**
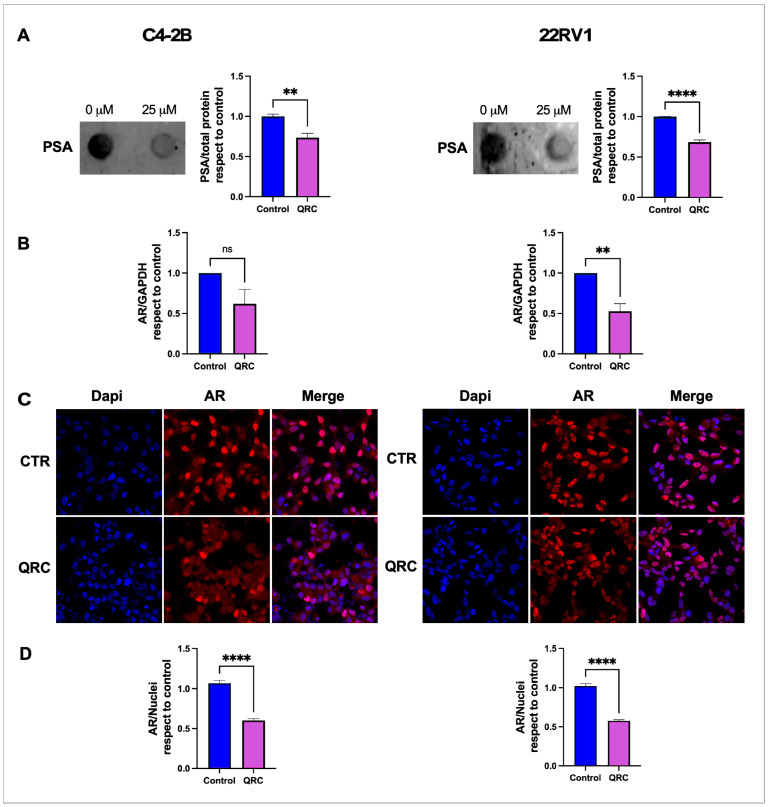
The effect of QRC on AR signaling in CRPC cell lines. (**A**) C4-2B and 22Rv1 cells were incubated in the presence or absence of 25 μM QRC for 24 h, and the culture medium was used for the analysis of the levels of PSA by dot blot. PSA was quantified after normalization against the concentration of total proteins in the medium, and data were plotted. (**B**) The real-time RT-PCR analysis of AR mRNA expression in C4-2B and 22Rv1 cells in response to 25 μM QRC for 24 h. (**C**) Representative images of the confocal analysis of the effect of QRC on AR nuclear distribution using DAPI as the nuclear marker. (**D**) For quantification, the intensity of the AR nuclear signal was normalized against DAPI. The data represent the means ± SEM of at least three independent experiments analyzed by Student’s *t*-test (**** *p* ≤ 0.0001, ** *p* ≤ 0.01). ns: not statistically significant.

The AR is a nuclear receptor, and therefore it must translocate to the nucleus to exert gene regulatory effects, such as the induction of the expression of the prostate-specific antigen (PSA) [39]. We investigated the effect of treatment with 25 μM QRC for 24 h on the levels of secreted PSA using dot blot and the nuclear localization of AR by immunocytochemical/confocal analysis. Remarkably, QRC reduced the levels of PSA by ~25 in C4-2B and by ~30% in 22Rv1 cell lines (Figure 4A). Given that AR signaling modulates AR expression as well, we assessed QRC’s effect on AR levels by real-time RT-PCR. Although AR mRNA levels were reduced by ~50% in both cell lines, statistical significance was only reached in 22Rv1 cells (Figure 4B). This could be explained, at least in part, by the reduction in the nuclear AR signal induced by QRC in both cell lines (Figure 4C), which reached ~50% (Figure 4D). Therefore, QRC acts as a negative modulator of the key AR signaling event, i.e., its nuclear accumulation, which affects critical targets, such as AR and PSA gene expression.

### 3.5. QRC and Enzalutamide Do Not Show Additive Anti-Proliferative Effects on CRPC Cell Lines at 48 h

**Figure 5:**
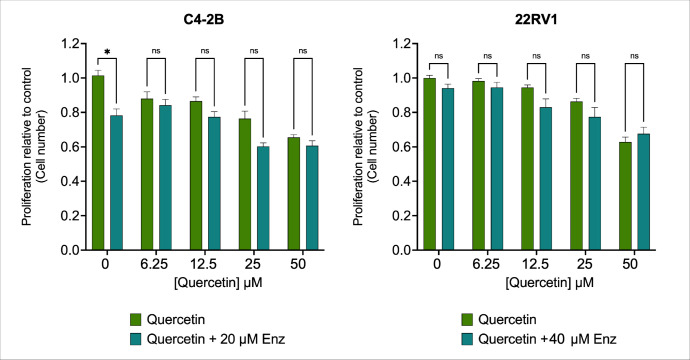
Enzalutamide (Enz) does not enhance the anti-proliferative effect of QRC on CRPC cell lines. C4-2B cells were cultured with 20 μM Enz and 22Rv1 cells with 40 μM Enz, and both cell lines were treated with increasing concentrations of QRC (0, 6.25, 12.5, 25 and 50 μM) up to 48 h to establish any additive effect. The plots show the effect of QRC alone (green bars) and the effect of Enz plus QRC (blue bars) after 48 h of treatment. The data represent the means ± SEM of at least three independent experiments analyzed by Student’s *t*-test (* *p* ≤ 0.05; ns: not statistically significant).

Enzalutamide (Enz) is an anti-androgen that prevents the AR from initiating gene transcription and promoting cell growth [25]. Enz inhibits the AR at multiple levels, preventing androgen binding to the receptor, inhibiting AR nuclear translocation, and interfering with AR-DNA interaction [40]. Because we had already shown that QRC suppresses AR signaling via the inhibition of the AKT–AR axis, we used Enz as a mechanistic comparator to test whether QRC acts upstream of canonical AR antagonism. Under this model, Enz would be expected to provide limited additional anti-proliferative benefit because AR activity is already constrained by QRC. Therefore, we tested the combined effect of Enz and QRC on the cell proliferation of CRPC cell lines using IncuCyte live-cell analysis during the 48 h treatment. Compared to C4-2B cells, 22Rv1 cells exhibit greater resistance to Enz, primarily due to the higher expression level of AR variants [25]; thus, we used 20 μM Enz for C4-2B cells and 40 μM Enz for 22Rv1. In our hands, while both QRC and Enz exerted cytostatic effects individually, no additive effects were observed after 48 h of co-incubation in either cell line (Figure 5).

## 4. Discussion

Antioxidants and redox-modulating phytochemicals are frequently discussed as ROS scavengers; however, many polyphenols also act as pleiotropic regulators of redox-sensitive signaling [41]. Our data provide a mechanistic hypothesis by which a redox-active natural product can modulate the AKT-AR axis through an allosteric pocket in AKT1. This target engagement framework is compatible with global changes in ROS, but it is not necessarily dependent on them. Because ROS-driven PTEN oxidation is a recognized route to amplify PI3K AKT signaling [42], QRC’s antioxidant activity could, in principle, converge with direct AKT engagement to dampen AKT activation. Similarly, AR phosphorylation and nuclear trafficking are influenced by broader signaling and stress adaptation programs that are intertwined with cellular redox status [43]. This dual framing, which includes direct allosteric inhibition and a potential dependence on redox context, may help interpret the breadth of QRC’s reported anticancer activities. In this sense, our results demonstrate that QRC exerts a cytostatic rather than cytotoxic effect on CRPC cell lines, in contrast to reports in androgen-sensitive LNCaP cells where QRC induces apoptosis, through a mechanism dependent on the reduced expression of hnRNPA1 and AR-V7, rather than a ROS-scavenging mechanism [44]. This distinction between androgen-dependent and androgen-independent PCa cells is relevant because androgen-independent subclones of LNCaP also showed variable sensitivity to QRC depending on AR pathway status [45]. Thus, the cytostatic effect observed in CRPC may reflect the QRC-mediated modulation of proliferative signaling without overt cell death within the time frame assessed, consistent with androgen-deprived survival programs that increase the apoptotic threshold through the upregulation of anti-apoptotic family proteins and support progression toward an androgen-independent, CRPC-like state [46,47]. A consistent finding was the reduction in AKT phosphorylation at S473 following QRC treatment. This phosphorylation site is required for full AKT activation [9,10], and its inhibition has been linked to impaired prostate tumor growth in vivo [15]. Our molecular docking and molecular dynamic analyses suggest that QRC interacts with a druggable pocket located in the N-LOBE of the AKT1 kinase domain. This binding mode proposes an allosteric mechanism of inhibition, supported by the stability of the QRC-AKT1 complex and the predicted contribution of residue G159 in ligand stabilization. Importantly, this pocket appears to differ from the canonical binding site of other allosteric inhibitors such as MK-2206, which depend on the W80 residue [48]. Consistent with this observation, our in silico mutagenesis analysis suggests that QRC may retain binding affinity even in the W80A/C variants, pointing to a potentially distinct interaction mode. However, these findings are derived from computational modeling and should therefore be interpreted as structural hypotheses rather than experimentally validated mechanisms. In particular, the predicted role of residue G159 in stabilizing QRC binding remains to be confirmed experimentally. Functional validation through the site-directed mutagenesis of G159, followed by binding or activity assays, will be necessary to determine its contribution to ligand recognition and inhibition. Although these experiments were beyond the scope of the present study, they represent an important direction for future work aimed at validating the proposed interaction mechanism between QRC and AKT1. Notably, this structural hypothesis is consistent with our preliminary unpublished observations showing that quercetin reduced AKT Thr308 phosphorylation in C4-2B cells and with the experimental findings of the present study demonstrating that quercetin decreases AKT Ser473 phosphorylation, a modification required for full AKT activation. Together, these observations support a potential inhibitory effect of QRC on AKT signaling.

We further observed that QRC reduced AR phosphorylation at S210/213, a modification associated with AR stabilization and nuclear translocation [49,50], and also decreased total AR levels after 24 h of treatment. These findings are consistent with previous reports in Enz-resistant models, where QRC reduced AR and AR-V7 expression through the regulation of hnRNPA1 [25]. Together, our data support a complementary mechanism whereby QRC suppresses AR signaling indirectly via AKT inhibition. This dual targeting is particularly relevant because AR overexpression and splice variant activation are central drivers of resistance in CRPC [51,52]. Furthermore, differences between transformed and non-transformed cell models [53] suggest that QRC selectively impairs tumor AR/AKT signaling while sparing normal cells.

The reciprocal regulation between AR and PI3K/AKT signaling has been widely documented [16,18]. While androgens can rescue apoptosis induced by PI3K inhibitors [12], PI3K/AKT activation can promote AR activity in the absence of ligands [19]. This crosstalk explains why monotherapy with either AR or AKT inhibitors often fails in clinical trials [8]. Our findings that QRC inhibits AKT phosphorylation, prevents AR nuclear localization and reduces PSA secretion further reinforce the concept that the simultaneous modulation of both pathways is necessary to restrain CRPC progression.

We observed that Enz alone showed limited efficacy in CRPC cells, particularly in 22Rv1. Furthermore, the combination of Enz and QRC did not produce an enhanced anti-proliferative effect or increased toxicity, even at high QRC doses. The lack of additive effects suggests that QRC already prevents AR activation upstream by reducing AR phosphorylation and nuclear localization, thereby limiting the incremental benefit of Enz. This contrasts with long-term clonogenic assays where QRC resensitized Enz-resistant CRPC cells [25], highlighting that treatment duration and cellular context may strongly influence the outcome. Clinical trials combining Enz with capivasertib, an ATP-competitive AKT inhibitor, have shown safety and tolerability but no significant improvement in efficacy [54], underscoring the need for more selective AKT modulators. Our data suggest that QRC, through its unique allosteric binding, may fulfill this role.

Despite these promising effects, the clinical use of QRC is limited by its poor solubility and low bioavailability [21]. Structural derivatives, such as QRC-7-O-β-glucopyranoside, have shown improved solubility while retaining AKT-inhibitory activity [55]. Delivery systems such as nanoemulsions have also enhanced the absorption and stability of QRC in preclinical models, providing a strategy to overcome pharmacokinetic limitations [21]. Altogether, QRC emerges as a promising compound that targets multiple nodes of the PI3K/AKT/AR axis: it binds to a novel allosteric pocket in AKT, reduces AR expression and phosphorylation, and prevents AR nuclear accumulation. This multifaceted mechanism may help overcome compensatory signaling, delay the onset of resistance, and support the use of natural products as scaffolds for the development of new drugs for CRPC.

## 5. Conclusions

In this study, we demonstrate that quercetin (QRC), a redox-active dietary flavonol, exerts predominantly cytostatic effects in CRPC cell models while consistently attenuating the AKT-AR signaling axis. In C4-2B and 22Rv1 cells, QRC decreased AKT activation and reduced AR phosphorylation, which was accompanied by lower AR protein abundance, impaired AR nuclear accumulation, and diminished PSA secretion. The absence of additive anti-proliferative activity upon cotreatment with enzalutamide is consistent with QRC acting upstream of ligand-driven AR activation under the conditions tested. Importantly, molecular docking and molecular dynamic simulations supported a plausible non-ATP, allosteric interaction between QRC and an N-lobe pocket in AKT1, providing a mechanistic framework distinct from classical ATP-competitive AKT inhibitors. Collectively, these results position QRC as a mechanistically informed natural product scaffold for targeting AKT-dependent AR signaling in CRPC and motivate further studies integrating redox biomarkers and additional pharmacological contexts to clarify the contribution of redox modulation and optimize translational relevance.

## Data Availability

All scripts and workflows of the molecular docking and molecular dynamic analyses are openly available in a dedicated GitHub repository (https://github.com/Sazocar-P/Molecular_docking_AKT_QUE.git (accessed on 28 May 2025)). To ensure reproducibility and long-term accessibility, this repository was archived in Zenodo under the DOI https://doi.org/10.5281/zenodo.15537643. All other data is available from the corresponding author upon request.

## Supplementary Materials

The following supporting information can be downloaded at https://www.mdpi.com/article/10.3390/antiox15030393/s1, Supplementary Figure S1. The quality of the selected 6BUU structural model. Supplementary Figure S2. The docking pose selection workflow for the quercetin-AKT1 complex. Supplementary Figure S3. The energy minimization of the AKT1–quercetin system. Supplementary Figure S4. The heating phase of the molecular dynamic simulation. Supplementary Figure S5. The equilibration of the AKT1–quercetin system. Supplementary Figure S6. Production molecular dynamic trajectory. Supplementary Figure S7. Dose–response curves from crystal violet assays. Representative Incucyte videos for proliferation and cell death assays can be found in the Supplementary Information.

## Abbreviations

The following abbreviations are used in this manuscript:

PCa	Prostate cancer
CRPC	Castration-resistant prostate cancer
AR	Androgen receptor
ADT	Androgen deprivation therapy
PSA	Prostate-specific antigen
PI3K	Phosphoinositide 3-kinase
AKT	Protein kinase B
AKT1	Protein kinase B alpha (AKT isoform 1)
PTEN	Phosphatase and tensin homolog
ROS	Reactive oxygen species
RNS	Reactive nitrogen species
NRF2	Nuclear factor erythroid 2-related factor 2
oxLDL	Oxidized low-density lipoprotein
NF-κB	Nuclear factor kappa-light-chain-enhancer of activated B cells
STAT3	Signal transducer and activator of transcription 3
QRC	Quercetin
Enz	Enzalutamide
DHT	Dihydrotestosterone
DMSO	Dimethyl sulfoxide
FBS	Fetal bovine serum
PBS	Phosphate-buffered saline
SDS-PAGE	Sodium dodecyl sulfate–polyacrylamide gel electrophoresis
RIPA	Radioimmunoprecipitation assay buffer
BCA	Bicinchoninic acid
TBS	Tris-buffered saline
BSA	Bovine serum albumin
DAPI	4′,6-diamidino-2-phenylindole
RT-PCR	Real-time reverse transcription PCR
PDB	Protein Data Bank
RMSD	Root mean square deviation
RMSF	Root mean square fluctuation
PCA	Principal component analysis
MD	Molecular dynamics
NPT	Constant number of particles, pressure, and temperature (ensemble)
