# Supplementary material for: Quercetin Inhibits AKT Ser473 Phosphorylation and Disrupts AKT–Androgen Receptor Signaling in Castration-Resistant Prostate Cancer Cells
Source: Antioxidants (Basel). 2026 Mar 20;15(3):393. doi: 10.3390/antiox15030393 (PMC13024314; doi:10.3390/antiox15030393)

A B

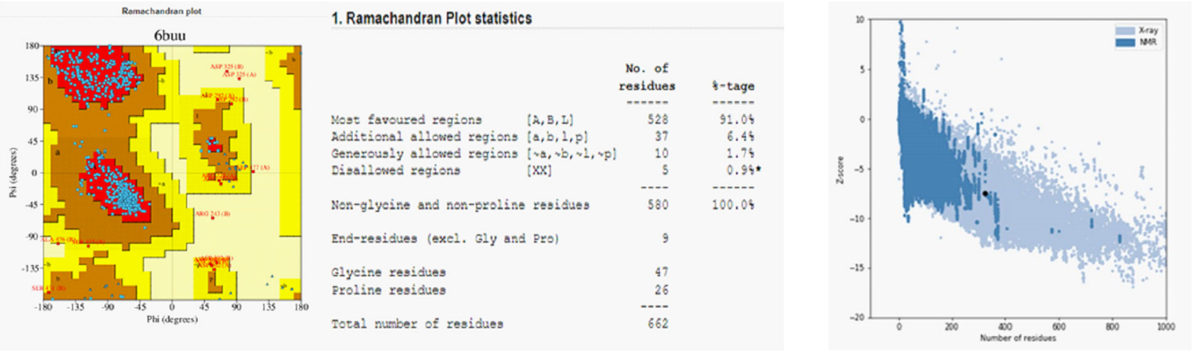

Supplementary Figure S1. ***Quality assessment of the selected 6BUU structural model***. (A) Ramachandran plot showing the distribution of phi and psi dihedral angles for residues in the 6BUU model. (B) Dispersion plot (Z-score: -7.4) illustrating the relationship between residue count and twist values, confirming the overall structural consistency of the model.

A B

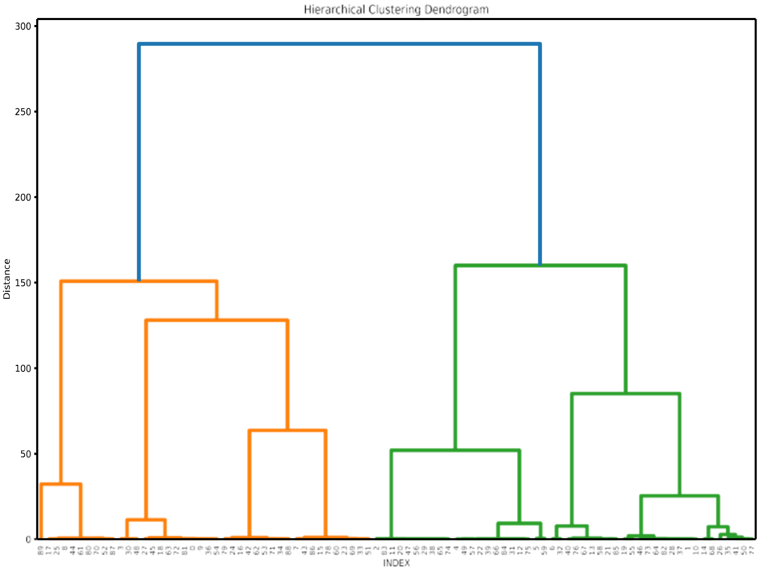

		10
45		
		19
14		

Supplementary Figure S2. ***Docking pose selection workflow for the quercetin–AKT1 complex***. **A** total of 90 independent docking simulations were performed. The optimal pose (QRC-14) was selected based on hierarchical clustering of RMSD values, proximity to the cluster centroid (A), and cluster population density (**B**).

Energy minimization steps in molecular dynamics

Minimization 1

```
AUH: initial minimization solvent + ions
 &cntrl
  imin   = 1,
  ntmin  = 1,
  maxcyc = 5000,
  ncyc   = 4000,
  ntb    = 1,
  ntr    = 1,
  cut    = 10.0,
  ntx    = 1,
  restraintmask = ':1-481',
  restraint_wt  = 500.0,
 /
```

Minimization 2

```
AUH: initial minimization hydrogen
 &cntrl
  imin          = 1,
  ntmin         = 1,
  maxcyc        = 10000,
  ncyc          = 8000,
  ntb           = 1,
  ntr           = 1,
  cut           = 10.0,
  ntx           = 1,
  restraintmask = ':1-481 & !@H=',
  restraint_wt  = 500.0,
 /
```

Minimization 3

```
AUH: intermediate minimizatior
 &cntrl
  imin           = 1,
  ntmin          = 1,
  maxcyc         = 10000,
  ncyc           = 5000,
  ntb            = 1,
  ntr            = 0,
  cut            = 10.0,
  ntxo           = 2,
  ntx            = 1,
  !restraintmask = '@N,CA,C',
  !restraint_wt  = 0.0,
 /
```

Minimization 4

```
AUH: minimization with flexible backbone restraints
 &cntrl
  imin          = 1,
  ntmin         = 2,
  maxcyc        = 20000,
  ncyc          = 10000,
  ntb           = 1,
  ntr           = 1,
  cut           = 10.0,
  ntxo          = 2,
  ntx           = 1,
  restraintmask = '@N,CA,C',
  restraint_wt  = 5.0,
 /
```

Minimization 5

```
AUH: minimization with different algorithm
 &cntrl
  imin          = 1,
  ntmin         = 2,
  maxcyc        = 20000,
  ncyc          = 10000,
  ntb           = 1,
  ntr           = 1,
  cut           = 12.0,
  ntxo          = 2,
  ntx           = 1,
  restraintmask = '@N,CA,C,O',
  restraint_wt  = 5.0,
 /
```

Minimization 6

```
AUH: minimization with experimental settings
 &cntrl
  imin          = 1,
  ntmin         = 2,
  maxcyc        = 10000,
  ncyc          = 5000,
  ntb           = 1,
  ntr           = 1,
  cut           = 10.0,
  ntxo          = 2,
  ntx           = 1,
  restraintmask = '@N,CA,C,O',
  restraint_wt  = 2.0,
 /
```

Minimization 7

```
AUH: final minimization before heating
 &cntrl
  imin   = 1,
  ntmin  = 2,
  maxcyc = 20000,
  ncyc   = 10000,
  ntb    = 1,
  cut    = 10.0,
  ntx    = 1,
  ntr    = 0,
  ntc    = 2,
  ntf    = 2,
 /
```

Supplementary Figure S3. ***Energy minimization of the AKT1–quercetin system***. Stepwise minimization was performed to relieve steric clashes and optimize the initial geometry of the system. The procedure included restrained minimization of solvent molecules followed by full-system minimization until a stable potential energy minimum was reached.

Heating steps in molecular dynamics

```
AUH: gradual heating from 0 K to 310 K
 &cntrl
  imin   = 0,
  irest  = 0,
  ntx    = 1,
  cut    = 10.0,
  ntb    = 1,
  ntr    = 1,
  ntc    = 2,
  ntf    = 2,
  ntt    = 3,
  tempi  = 0.0,
  temp0  = 310.0,
  gamma_ln = 1.0,
  nstlim = 300000,
  dt     = 0.001,
  ntpr   = 1000,
  ntwx   = 1000,
  ntwr   = 1000,
  iwrap  = 1,
  ioutfm = 1,
  nmropt = 1,
  restraintmask = ':1-481',
  restraint_wt  = 10.0,
 /
 &wt type='TEMP0', istep1=0, istep2=300000, value1=0.0, value2=310.0 /
 &wt type='END' /
 &wt type='REST', istep1=0, istep2=100000, value1=10.0, value2=1.0 /
 &wt type='REST', istep1=100001, istep2=200000, value1=1.0, value2=0.1 /
 &wt type='REST', istep1=200001, istep2=300000, value1=0.1, value2=0.0 /
 &wt type='END' /
```

Supplementary Figure S4. ***Heating phase of the molecular dynamics simulation.*** The system was gradually heated from 0 K to 300 K under an NVT ensemble using a Langevin thermostat. Positional restraints were applied to the protein backbone to prevent structural distortions during temperature equilibration.

Equilibrium steps in molecular dynamics

Equilibrium 1

```
200ps MD
 &cntrl
  imin = 0, irest = 1, ntx = 5,
  ntb = 2, pres0 = 1.0, ntp = 1,
  taup = 2.0,
  cut = 10.0, ntr = 1,
  ntc = 2, ntf = 2,
  ig= -1,
  temp0 = 310,
  ntt = 3, gamma_ln = 1.0,
  ioutfm = 1,
  iwrap  = 1,
  nstlim = 100000, dt = 0.001,
  ntpr = 10000, ntwx = 10000, ntwr = 10000,
  restraintmask = ':1-481',
  restraint_wt  = 10.0,
  /
```

Equilibrium 2

```
2000ps MD
 &cntrl
  imin = 0, irest = 1, ntx = 5,
  ntb = 2, pres0 = 1.0, ntp = 1,
  taup = 2.0,
  cut = 10.0, ntr = 1,
  ntc = 2, ntf = 2,
  ig= -1,
  temp0 = 310,
  ntt = 3, gamma_ln = 5.0,
  ioutfm = 1,
  iwrap  = 1,
  nstlim = 1000000, dt = 0.001,
  ntpr = 1000, ntwx = 1000, ntwr = 10000,
  restraintmask = ':1-481',
  restraint_wt  = 5.0,
  /
```

Equilibrium 3

```
&cntrl
  imin = 0,
  irest = 1,
  ntx = 5,
  ntb = 2,
  pres0 = 1.0,
  ntp = 1,
  taup = 2.0,
  cut = 10.0,
  ntr = 1,
  ntc = 2,
  ntf = 2,
  ig = -1,
  temp0 = 310,
  ntt = 3,
  gamma_ln = 1.0,
  ioutfm = 1,
  iwrap = 1,
  nstlim = 1000000,
  dt = 0.001,
  ntpr = 1000,
  ntwx = 1000,
  ntwr = 10000,
  restraintmask = ':1-481',
  restraint_wt = 1.0,
/
```

Equilibrium 4

```
&cntrl
  imin = 0,
  irest = 1,
  ntx = 5,
  ntb = 2,
  pres0 = 1.0,
  ntp = 1,
  taup = 2.0,
  cut = 10.0,
  ntr = 1,
  ntc = 2,
  ntf = 2,
  ig = -1,
  temp0 = 310,
  ntt = 3,
  gamma_ln = 1.0,
  ioutfm = 1,
  iwrap = 1,
  nstlim = 1000000,
  dt = 0.001,
  ntpr = 1000,
  ntwx = 1000,
  ntwr = 10000,
  restraintmask = ':1-481',
  restraint_wt = 0.1,
/
```

Equilibrium 5

```
&cntrl
  imin = 0,
  irest = 1,
  ntx = 5,
  ntb = 2,
  pres0 = 1.0,
  ntp = 1,
  taup = 2.0,
  cut = 10.0,
  ntr = 1,
  ntc = 2,
  ntf = 2,
  ig = -1,
  temp0 = 310,
  ntt = 3,
  gamma_ln = 5.0,
  ioutfm = 1,
  iwrap = 1,
  nstlim = 1000000,
  dt = 0.001,
  ntpr = 1000,
  ntwx = 1000,
  ntwr = 5000,
  restraintmask = ':1-481',
  restraint_wt = 0.05,
/
```

Equilibrium 6

```
&cntrl
  imin = 0,
  irest = 1,
  ntx = 5,
  nstlim = 1000000,
  dt = 0.001,
  ntb = 2,
  pres0 = 1.0,
  ntp = 1,
  taup = 2.0,
  cut = 10.0,
  ntr = 0,
  ntc = 2,
  ntf = 2,
  ig = -1,
  temp0 = 310,
  ntt = 3,
  gamma_ln = 5.0,
  ioutfm = 1,
  iwrap = 1,
  ntpr = 1000,
  ntwx = 1000,
  ntwr = 1000,
  /
```

Supplementary Figure S5. *Equilibration of the AKT1–quercetin system.* Equilibration was carried out under an NPT ensemble to stabilize pressure and density. Backbone restraints were progressively released to allow system relaxation while maintaining structural integrity before initiating production dynamics.

Production steps in molecular dynamics

Production 1

```
&cntrl
  imin = 0,
  nstlim = 100000000,
  dt = 0.001,
  irest = 1,
  ntx = 5,
  ig = -1,
  temp0 = 310.0,
  ntc = 2,
  ntf = 2,
  tol = 0.00001,
  ntwx = 1000,
  ntwe = 0,
  ntwr = 1000,
  ntpr = 1000,
  cut = 10.0,
  iwrap = 0,
  ntt = 3,
  gamma_ln = 1.0,
  ntb = 2,
  ntp = 1,
  nscm = 1000,
  barostat = 2,
  ioutfm = 1,
  ntxo = 2,
/
```

Production 2

```
&cntrl
  imin = 0,
  nstlim = 100000,
  dt = 0.001,
  irest = 1,
  ntx = 5,
  ig = -1,
  temp0 = 310.0,
  ntc = 2,
  ntf = 2,
  tol = 0.00001,
  ntwx = 20000,
  ntwe = 0,
  ntwr = 20000,
  ntpr = 20000,
  cut = 10.0,
  iwrap = 0,
  ntt = 3,
  gamma_ln = 1.0,
  ntb = 2,
  ntp = 1,
  nscm = 20000,
  barostat = 2,
  ioutfm = 1,
  ntxo = 2,
/
```

Production 3

```
&cntrl
  imin = 0,
  nstlim = 100000000,
  dt = 0.001,
  irest = 1,
  ntx = 5,
  ig = -1,
  temp0 = 310.0,
  ntc = 2,
  ntf = 2,
  tol = 0.00001,
  ntwx = 50000,
  ntwe = 0,
  ntwr = 50000,
  ntpr = 10000,
  cut = 10.0,
  iwrap = 0,
  ntt = 3,
  gamma_ln = 1.0,
  ntb = 2,
  ntp = 1,
  nscm = 10000,
  barostat = 2,
  ioutfm = 1,
  ntxo = 2,
/
```

Supplementary Figure S6. ***Production molecular dynamics trajectory.*** A 100 ns production simulation was performed under stable NPT conditions without restraints. This trajectory was used for all structural, dynamical, and interaction analyses reported in this study.

Supplementary Methods—Crystal Violet Assay and IC50 Analysis

C4-2B and 22Rv1 cells were seeded in 96-well plates (5,000 cells per well) and treated for 48 h with quercetin at 0, 6.25, 12.5, 25, 50, and 100 μM (vehicle control: 0.1% v/v DMSO). After treatment, the wells were washed with PBS, fixed with 4% PFA, stained with 0.1% (w/v) crystal violet, extensively rinsed, air-dried, and the dye was solubilized in 10% acetic acid. Absorbance was measured at 590/595 nm. Values were background-corrected and normalized to the vehicle control. IC50 values were estimated by nonlinear regression using a four-parameter logistic model (variable slope) and are reported with 95% confidence intervals from the nonlinear fit. Analyses were performed in GraphPad Prism 10. A p value < 0.05 was considered statistically significant when applicable. Four independent biological replicates were analyzed.

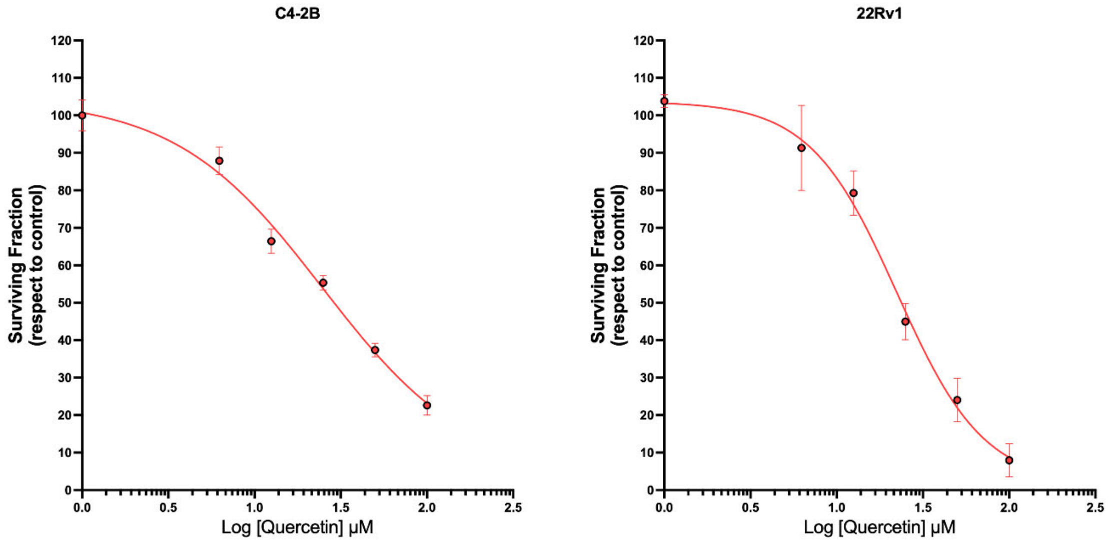

Supplementary Figure S7. Dose–response curves from crystal violet assays. Dose–response curves were fitted by nonlinear regression using a four-parameter logistic model (variable slope), and IC50 values were reported with 95% confidence intervals. The lowest QRC concentration tested showed no effect compared with the control. Therefore, the half-maximal growth inhibitory concentration (IC50) was estimated from the proliferation curves as 24.37 μM for C4-2B cells and 21.54 μM for 22Rv1 cells.

Supplementary Video Links:

Supplementary video S1_C4-2B Control DMSO 0.1%:

https://drive.google.com/file/d/1JPu_oKYrlILGniWvMAIlZc7wEYqwuEn4/view?usp=drive_link

Supplementary video S2_C4-2B QRC 25uM:

https://drive.google.com/file/d/1ML0uCvmHQLxfdsjCqpNpBJW_z_xF_S8q/view?usp=drive_link

Supplementary video S3_C4-2B QRC 50uM

https://drive.google.com/file/d/1ANsJtOeqdPh-r_cjVyeAji29F_CTMARq/view?usp=drive_link

Supplementary video S4_22rv1 Control DMSO 0.1%

https://drive.google.com/file/d/10mC40fstoIXt2havJ5qAv54HlcwaMM-m/view?usp=drive_link

Supplementary video S5_22rv1 QRC 25 uM

https://drive.google.com/file/d/1f86gOoJ3_ujPPHFv_opUZYVYOLEfYMiM/view?usp=drive_link

Supplementary video S6_22rv1 QRC 50uM

https://drive.google.com/file/d/1Sg8SXOEb8lyQVImWIA1IROsjJuTrAGr-/view?usp=drive_link

Supplementary Original Immunoblots

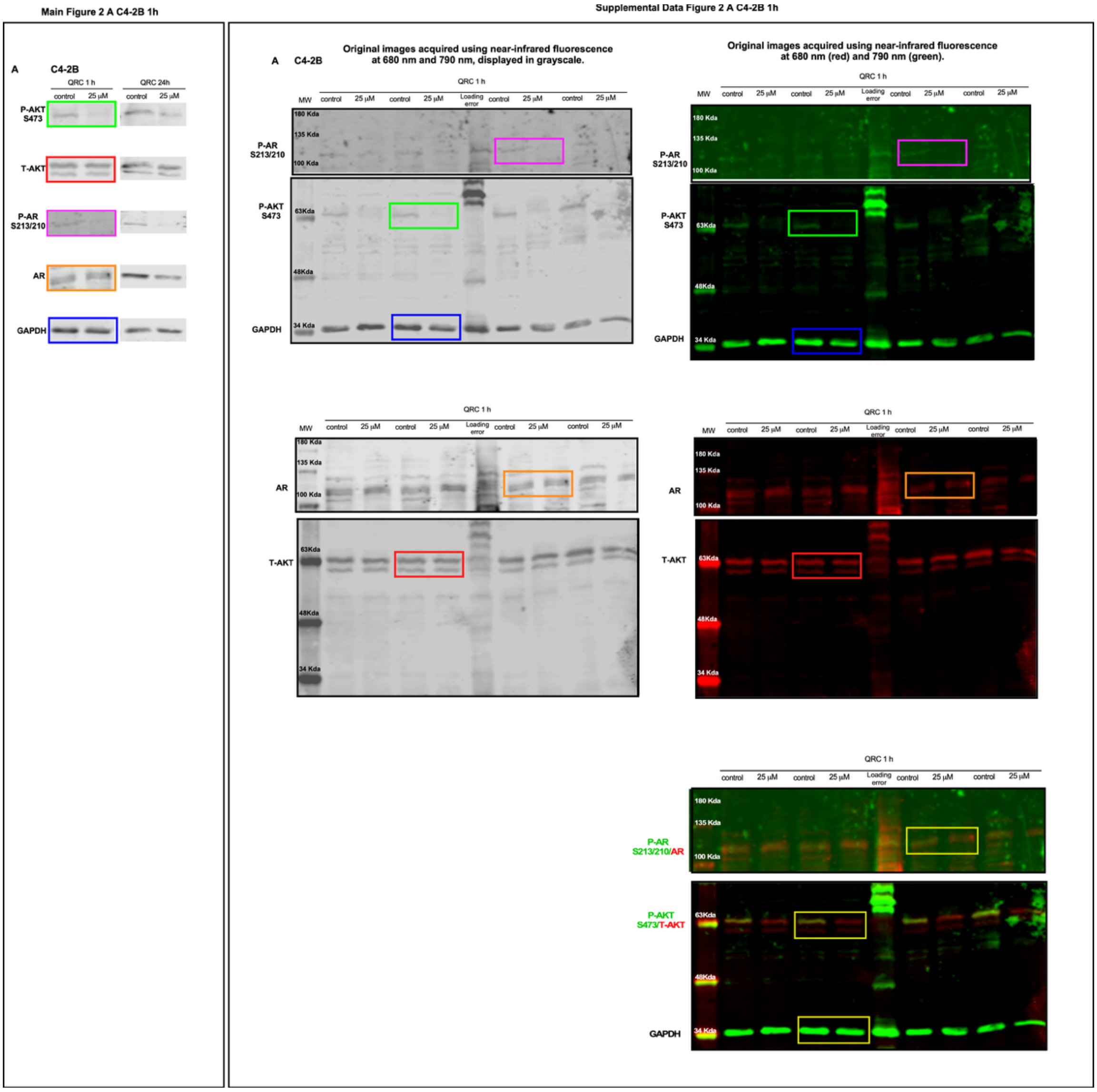

Supplemental Data Figure 2 A C4-2B 24h

Main Figure 2 A C4-2B 24h

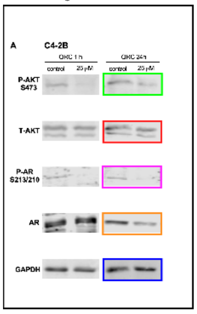

Original Images acquired using near-infrared fluorescence at 680 nm and 790 nm, displayed in grayscale.

Original images acquired using near-infrared fluorescence at 680 nm (red) and 790 nm (green).

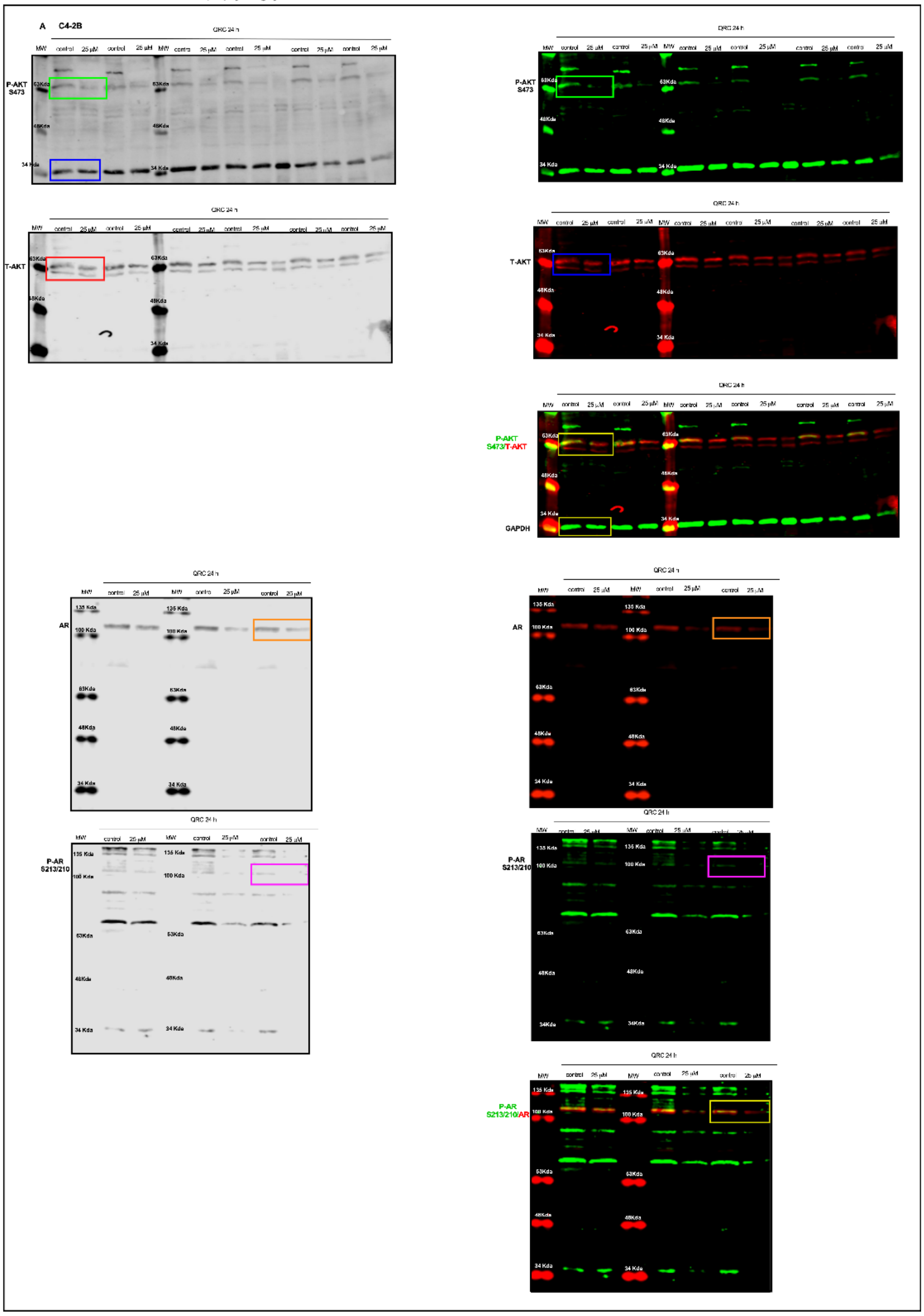

Main Figure 2 B 22Rv1 1h

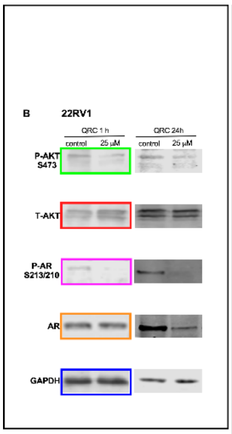

Supplemental Data Figure 2 B 22Rv1 1h

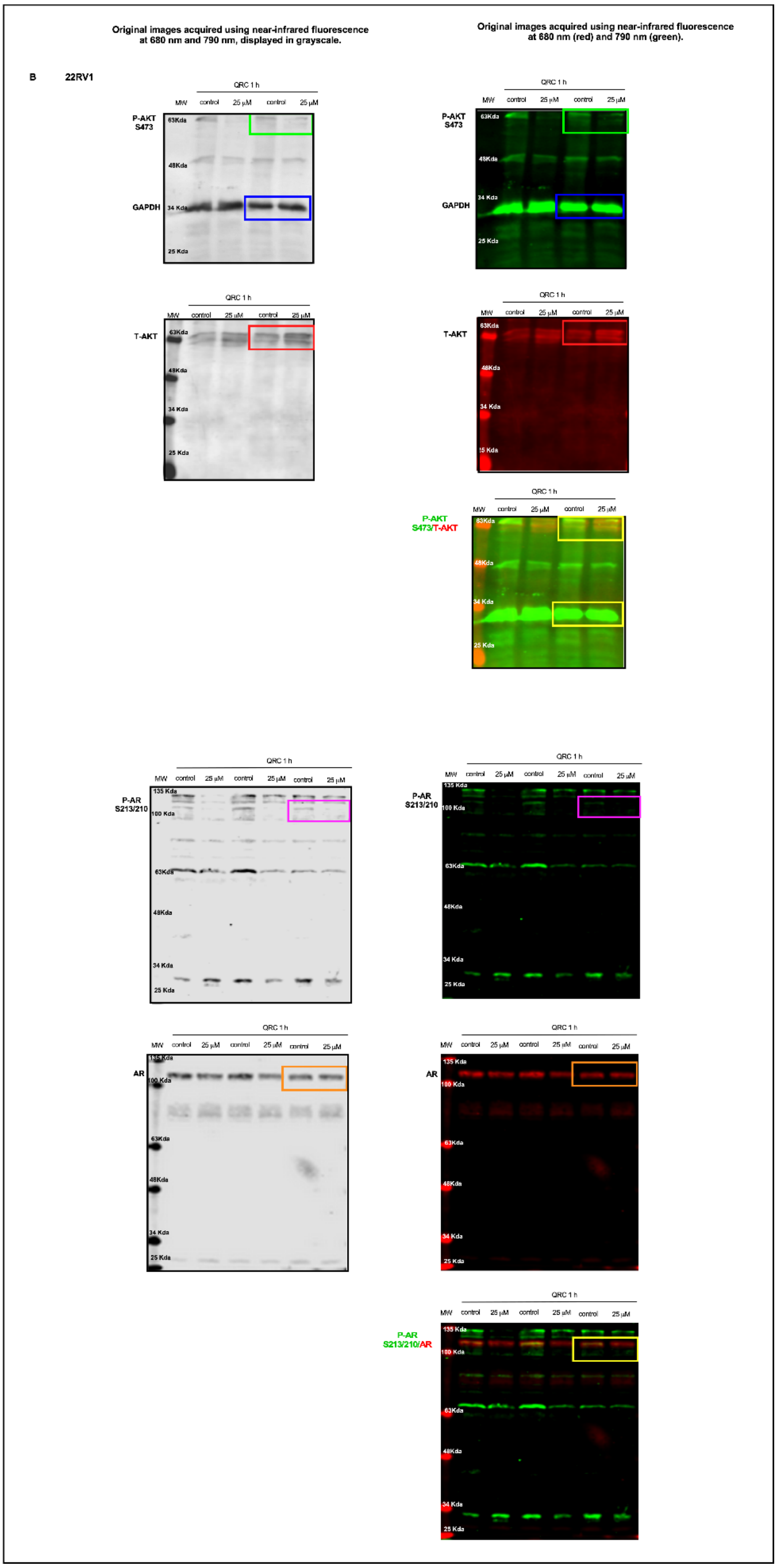

Main Figure 2 B 22Rv1 24h

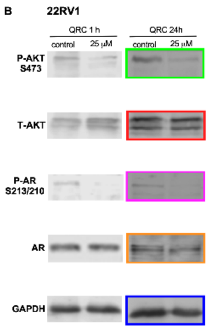

Supplemental Data Figure 2 B 22Rv1 24h

Original images acquired using near-infrared fluorescence at 680 nm and 790 nm, displayed in grayscale.

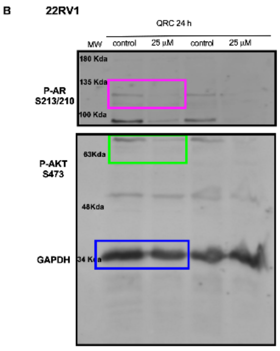

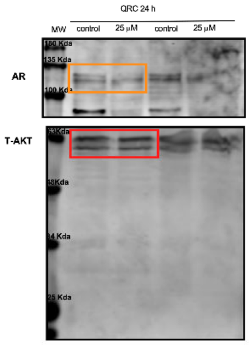

Original images acquired using near-infrared fluorescence at 680 nm (red) and 790 nm (green).

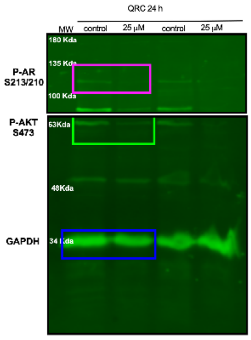

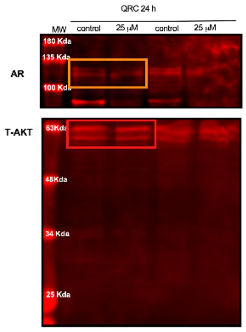

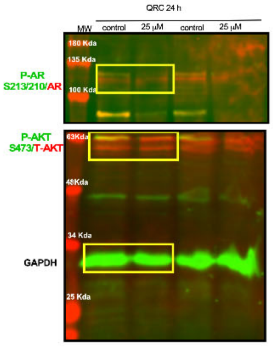

Main Figure 4 A

Supplemental Data Figure Figure 4A

Figure 4

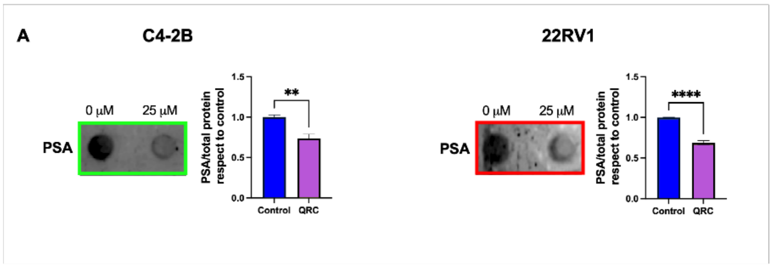

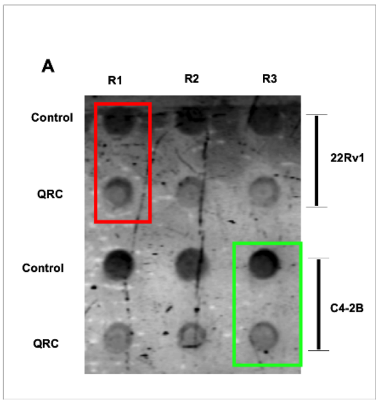

Supplement: Supplementary file 1 [file antioxidants-15-00393-s001.zip › antioxidants-4149254-supplementary.pdf]
